# Supplementary material for: Comparison of unbiased metagenomic next generation sequencing to targeted multiplex diagnostic assays for the detection of respiratory viruses
Source: PLoS One. 2026 May 7;21(5):e0347750. doi: 10.1371/journal.pone.0347750 (PMC13152136; doi:10.1371/journal.pone.0347750)
Supplement: S1 Fig — (DOCX) [file pone.0347750.s001.docx]

**S1 Fig. Detection of SARS-CoV-2 reads across the dilution series**

**S1 Fig. Detection of SARS-CoV-2 reads across the dilution series:** A PCR positive SARS-CoV-2 NP sample diluted to create a dilution series ranging from 10⁶ to 10¹ copies/mL. The diluted samples were then analayzed by metagenomic next generation sequencing.
