# Supplementary material for: Comparison of unbiased metagenomic next generation sequencing to targeted multiplex diagnostic assays for the detection of respiratory viruses
Source: PLoS One. 2026 May 7;21(5):e0347750. doi: 10.1371/journal.pone.0347750 (PMC13152136; doi:10.1371/journal.pone.0347750)
Supplement: S1 Table — (DOCX) [file pone.0347750.s002.docx]

**S1 Table. Total and non-human read counts for controls and samples from cohort 1 and cohort 2 sequenced on the NovaSeq 6000**

| **Sample ID** | **Cohort** | **Total Reads** | **Non-Human Reads** |
| --- | --- | --- | --- |
| Control 1 | Control | 17,406,628 | 17,394,213 |
| Control 2 | Control | 16,907,296 | 16,895,565 |
| A1 | 1 | 3,389,793 | 2,642,482 |
| A2 | 1 | 11,727,388 | 9,980,882 |
| A3 | 1 | 25,774,838 | 20,933,318 |
| A4 | 1 | 12,816,017 | 8,852,427 |
| A5 | 1 | 36,210,738 | 24,932,251 |
| A6 | 1 | 13,499,008 | 13,085,529 |
| A7 | 1 | 24,508,673 | 17,897,783 |
| A8 | 1 | 28,662,849 | 25,962,909 |
| A9 | 1 | 27,958,646 | 22,653,658 |
| A10 | 1 | 23,279,629 | 20,186,117 |
| A11 | 1 | 30,035,841 | 29,250,853 |
| A12 | 1 | 40,966,059 | 38,403,397 |
| A13 | 1 | 10,192,105 | 10,128,839 |
| A14 | 1 | 5,253,022 | 5,036,317 |
| A15 | 1 | 4,529,710 | 4,384,497 |
| A16 | 1 | 5,013,878 | 4,823,735 |
| A17 | 1 | 14,531,259 | 14,144,889 |
| A18 | 1 | 8,684,242 | 8,244,756 |
| A19 | 1 | 16,418,380 | 14,876,385 |
| A20 | 1 | 9,955,995 | 9,600,102 |
| A21 | 1 | 9,502,171 | 9,098,657 |
| A22 | 1 | 4,664,445 | 4,371,385 |
| A23 | 1 | 6,230,875 | 5,117,303 |
| A24 | 1 | 5,987,621 | 5,355,417 |
| A25 | 1 | 13,583,081 | 12,927,024 |
| A26 | 1 | 3,311,262 | 3,244,879 |
| A27 | 1 | 7,245,303 | 6,771,225 |
| A28 | 1 | 5,457,248 | 5,457,013 |
| A29 | 1 | 14,293,512 | 13,933,559 |
| A30 | 1 | 28,109,369 | 23,311,762 |
| A31 | 1 | 24,839,592 | 16,918,541 |
| A32 | 1 | 20,696,118 | 16,838,263 |
| A33 | 1 | 23,416,989 | 18,342,955 |
| A34 | 1 | 20,992,106 | 20,124,205 |
| A35 | 1 | 30,519,404 | 27,203,523 |
| A36 | 1 | 22,952,456 | 16,770,551 |
| A37 | 1 | 10,579,756 | 9,544,894 |
| A38 | 1 | 39,184,811 | 31,788,723 |
| A39 | 1 | 590,874 | 472,422 |
| A40 | 1 | 211,354 | 151,412 |
| A41 | 1 | 14,587,758 | 13,623,076 |
| A42 | 1 | 17,403,887 | 14,393,887 |
| A43 | 1 | 10,679,053 | 9,659,269 |
| A44 | 1 | 17,396,068 | 13,910,565 |
| A45 | 1 | 16,531,754 | 13,563,804 |
| A46 | 1 | 20,915,046 | 16,490,160 |
| A47 | 1 | 22,948,119 | 17,186,773 |
| A48 | 1 | 47,035,834 | 32,090,809 |
| A49 | 1 | 22,791,438 | 11,853,802 |
| A50 | 1 | 21,775,101 | 18,708,967 |
| A51 | 1 | 11,958,673 | 10,955,857 |
| A52 | 1 | 31,835,622 | 24,433,133 |
| Control 3 | Control | 31,329,938 | 31,312,149 |
| Control 4 | Control | 19,912,515 | 19,900,216 |
| A53 | 2 | 34,593,040 | 33,932,050 |
| A54 | 2 | 16,404,521 | 15,840,234 |
| A55 | 2 | 25,506,117 | 24,947,555 |
| A56 | 2 | 20,476,875 | 19,436,384 |
| A57 | 2 | 50,029,289 | 49,688,921 |
| A58 | 2 | 19,635,746 | 19,414,964 |
| A59 | 2 | 50,263,727 | 49,436,858 |
| A60 | 2 | 32,885,442 | 13,686,435 |
| A61 | 2 | 37,951,833 | 37,413,414 |
| A62 | 2 | 30,547,162 | 30,453,560 |
| A63 | 2 | 36,678,965 | 35,784,420 |
| A64 | 2 | 35,468,892 | 35,455,988 |
| A65 | 2 | 20,839,397 | 20,756,980 |
| A66 | 2 | 19,827,898 | 18,926,708 |
| A67 | 2 | 18,747,357 | 17,708,468 |
| A68 | 2 | 20,016,037 | 18,967,387 |
| A69 | 2 | 16,782,174 | 16,426,375 |
| A70 | 2 | 29,993,441 | 29,971,373 |
| A71 | 2 | 24,756,522 | 24,183,730 |
| A72 | 2 | 14,709,884 | 14,398,402 |
| A73 | 2 | 27,708,897 | 25,778,758 |
| A74 | 2 | 24,029,757 | 23,448,590 |
| A75 | 2 | 35,784,002 | 34,921,068 |
| A76 | 2 | 28,825,357 | 28,264,767 |
| A77 | 2 | 17,213,768 | 17,122,031 |
| A78 | 2 | 17,433,115 | 16,926,688 |
| A79 | 2 | 10,970,774 | 10,862,866 |
| A80 | 2 | 22,630,398 | 22,389,559 |
| A81 | 2 | 28,844,875 | 27,144,281 |
| A82 | 2 | 19,101,664 | 19,052,781 |
| A83 | 2 | 30,528,362 | 30,399,946 |
| A84 | 2 | 25,953,975 | 25,934,039 |
| A85 | 2 | 13,393,454 | 13,344,276 |
| A86 | 2 | 26,636,753 | 26,598,194 |
| A87 | 2 | 25,052,180 | 25,012,910 |
| A88 | 2 | 7,455,015 | 7,332,371 |
| A89 | 2 | 12,661,937 | 12,494,416 |
| A90 | 2 | 33,167,592 | 32,559,000 |
| A91 | 2 | 27,304,640 | 25,768,470 |
| A92 | 2 | 23,845,051 | 23,626,323 |
| A93 | 2 | 19,821,583 | 19,188,964 |
| A94 | 2 | 12,185,420 | 11,826,533 |
| A95 | 2 | 28,396,367 | 28,009,317 |
| A96 | 2 | 19,061,236 | 18,409,191 |
| A97 | 2 | 26,611,843 | 26,433,640 |
| A98 | 2 | 20,736,873 | 20,125,943 |
| A99 | 2 | 15,346,855 | 15,001,505 |
| A100 | 2 | 13,048,406 | 13,045,976 |
