# Supplementary material for: Comparison of unbiased metagenomic next generation sequencing to targeted multiplex diagnostic assays for the detection of respiratory viruses
Source: PLoS One. 2026 May 7;21(5):e0347750. doi: 10.1371/journal.pone.0347750 (PMC13152136; doi:10.1371/journal.pone.0347750)
Supplement: S2 Table — (DOCX) [file pone.0347750.s003.docx]

**S2 Table. mNGS results for samples evaluated by ePLEX RP**

| **Sample** | **mNGS Species Call** | **ePlex RP Result** |
| --- | --- | --- |
| A53 | ND | Rhinovirus/Enterovirus |
| A54 | Streptococcus pneumoniae | ND |
| A55 | ND | Rhinovirus/Enterovirus/metapneumovirus |
| A56 | Bacillus thermoamylovorans | ND |
| A57 | Dolosigranulum pigrum, Moraxella catarrhalis | ND |
| A58 | Human orthopneumovirus (RSV) | RSV B |
| A59 | ND | Influenza A H3 |
| A60 | ND | Influenza A H3 |
| A61 | ND | Influenza A H3 |
| A62 | ND | Rhinovirus/Enterovirus |
| A63 | ND | Adenovirus |
| A64 | ND | ND |
| A65 | ND | ND |
| A66 | Influenza A | Influenza A H1N1 |
| A67 | Bacillus thermoamylovorans | ND |
| A68 | ND | ND |
| A69 | Influenza A | Influenza A H1N1 |
| A70 | ND | Influenza A H1N1 |
| A71 | ND | ND |
| A72 | ND | ND |
| A73 | ND | ND |
| A74 | Acanthamoeba castellanii | Adenovirus |
| A75 | Human orthopneumovirus (RSV) | RSV B |
| A76 | Dolosigranulum pigrum | RSV A |
| A77 | ND | Rhinovirus/Enterovirus |
| A78 | ND | ND |
| A79 | ND | ND |
| A80 | ND | ND |
| A81 | ND | ND |
| A82 | ND | ND |
| A83 | Human parainfluenza virus 3 | Human parainfluenza virus 3 |
| A84 | Coronavirus NL63 | Coronavirus NL63 |
| A85 | ND | RSV A |
| A86 | ND | ND |
| A87 | Acanthamoeba castellanii | ND |
| A88 | Bacillus thermoamylovorans | ND |
| A89 | ND | ND |
| A90 | ND | ND |
| A91 | ND | ND |
| A92 | Coronavirus 229E | Coronavirus 229E |
| A93 | ND | Rhinovirus/Enterovirus |
| A94 | Influenza A | Influenza A H3 |
| A95 | ND | Metapneumovirus |
| A96 | Dolosigranulum pigrum, Moraxella catarrhalis | Adenovirus |
| A97 | ND | Parainfluenza virus 3 |
| A98 | Bacillus thermoamylovorans | Influenza A H1N1 |
| A99 | ND | Rhinovirus/Enterovirus |
| A100 | ND | ND |

ND: None Detected
