# Supplementary material for: Comparison of unbiased metagenomic next generation sequencing to targeted multiplex diagnostic assays for the detection of respiratory viruses
Source: PLoS One. 2026 May 7;21(5):e0347750. doi: 10.1371/journal.pone.0347750 (PMC13152136; doi:10.1371/journal.pone.0347750)
Supplement: Attachment — Submitted filename: eplex MNGS_PLOS_V1_reviewer_comments_030326.docx [file pone.0347750.s005.docx]

We thank the reviewer for their thoughtful and constructive comments. We have now incorporated these modifications into the revised version of the manuscript, and we believe they have helped us improve the clarity and impact of our study.

**Reviewer #1: Comments:**
1) The manuscript reports a comparison between two diagnostic approaches: multiplex PCR-based viral panels and metagenomic NGS, applied to two relatively small cohorts of approximately 50 samples each. It’s not clear why the authors chose specifically to compare between these two approaches, but completely neglect to mention sequencing-based probe panel approaches like TWIST, Illumina Viral Surveillance Panel v2, or the earlier ViroCap, which target a broader range of viruses than multiplex PCR panels, while being more focused and specific than untargeted metagenomic sequencing. This important category which is gaining popularity as a diagnostic assay is entirely overlooked, including in the introduction and discussion.

We appreciate the reviewer’s comment and have now revised the text to clarify the rational of our approach. We agree that **sequencing-based probe panels** target a broader range of viruses than multiplex PCR panels and **are** more focused and specific than untargeted metagenomic sequencing. However, **because these probe-based panels are not yet widely implemented in routine clinical laboratories**, we **chose to compare with the more commonly used FDA-cleared, multiplex platforms to allow for a robust and clinically relevant comparison.** mNGS has become popular with clinicians as an unbiased, hypothesis-free genomic interrogation of a clinical specimen without consideration of the relative sensitivity and performance (PMID:40870843, PMID:39533109). For example, it has been used in bronchoalveolar lavage and in sputum specimens from patients with lower respiratory tract infections and cerebrospinal fluid for suspected central nervous system infections(PMID:40142572;PMID:35695488). All of the nasopharyngeal (NP) swabs in viral transport media (VTM) used in our evaluation had been previously tested for microbial pathogens using commercially available **FDA-approved** multiplex molecular assays (Roche eplex). As the costs associated with sequencing decrease, we sought to test the concordance of mNGS on NP swabs in VTM with the commercial FDA-cleared multiplex PCR assay, and to assess whether mNGS would give additional detection ‘calls’ for symptomatic eplex negative participants given the high levels of sequence variations with respiratory viruses as well as other novel, emerging pathogens.

2) Overall the manuscript is not written well or very clear. The text is brief and lacks sufficient detail, making it difficult to follow. The current format resembles a short communication or brief report, and many parts are not clearly articulated. In several sections, explanations are omitted and substantial prior knowledge is assumed, which considerably impairs readability and interpretation. The manuscript would benefit from a thorough rewrite to improve clarity, expand on methodological details, and provide more explicit explanations of the results and their implications.

In response to the reviewer’s comments, we have substantially revised the manuscript to improve overall readability, provide more detailed methodological descriptions, and expand explanations of the results and their implications.

Other comments
1) I would mention what Xpert Xpress / ePlex RP2 are, it’s not very clear.

We have revised the Methods section to include additional details describing the Xpert Xpress CoV-2/Flu/RSV Plus and ePlex RP2 assays (lines 101-106, 107–115, 126), clarifying their function as commercially available, FDA-cleared, multiplex molecular diagnostic platforms.

2) this part “Participant median age was 40 years (IQR 31-51) with 68% (68/100) females” is more methods than results and maybe shouldn’t be included in the abstract.

We thank the reviewer for this comment. The statement describing participant age and sex distribution has been removed from the Abstract and relocated to the Methods section (lines 83).

3) Its not clear from the abstract that the Xpert Xpress (cohort 1) are the “negatives” that were evaluated with mNGS.

We thank the reviewer for this comment. The abstract has been revised to explicitly clarify that Cohort 1 consists of Xpert Xpress–negative samples that were subsequently evaluated by mNGS.

4) Line 47: fix the wording of the sentence

Thank you for the helpful suggestion. The introduction has been substantially revised for clarity and readability, and the sentence previously located at line 47 has been reworded and incorporated into the revised text

5) Line 57: sentence not so clear - what other samples? Is this concentration good? Compared to what?

We apologize for the lack of clarity. The sentence has been revised to specify the sample type and to clarify the concentration threshold relative to standard clinical assays (lines 68-70).

6)Line 70: missing details about the ePlex RP2 system*.*

Thank you for highlighting this. We have added an additional sentence to provide more details about the pathogens detected by the ePlex RP2 system (lines 109-113).

7) Line 70: “clinical characteristics..” add “and ePlex RP2 test results” (also to the table header)

We have now revised table 1 heading and the text (lines 92-93).

*8)* Line 86: define which of the controls you refer to later on as “control 1” and “control 2” (e.g. in Figure 1a)

Thank you for helping us improve the manuscript. The Acrometrix™ Multi-Analyte SARS-CoV-2, influenza A/B, and RSV A/B were assigned as controls 1-4 and this has now been clarified in the text (lines 130,157,177).

9) Line 95: how many reads were allocated per sample?

A median of 20,586,497 reads (IQR 14,78,026) was obtained from sequencing samples from Cohorts 1 and 2. This information has now been added to the revised manuscript on a cohort basis (lines 159-161,179-181). In addition, Supplementary Table 1 has been included to provide the read counts for each individual sample and control.

10) Line 107: why was the >50 reads threshold chosen? (out of how many reads?) is this a Kraken threshold, or a custom one?

There is no fixed read count threshold that is the same for each species. Instead, a z-score based statistical cutoff was used to establish a true species call, as implemented in Pavian (PMID:31553437). The smallest number of reads satisfying this cutoff in any sample was 50. For example, a read count of 25 reads for species A is more meaningful when there are 0 species A reads in every other sample. However, if all samples have an equal number of 25 species B reads, this is less meaningful for the analysis, leading to a more likely explanation for contamination during the collection or sequencing process. Accordingly, species detection was determined by z-scores to determine read counts that meet the limit of detection. We now mention this in the revised manuscript (lines 149-151).

11) Line 118: “some of the detected species are potentially pathogenic” – not clear what this means? By what measure?

We apologize for the lack of clarity. We have revised the text to define “potentially pathogenic” as organisms that are known to cause disease only under specific host conditions (e.g., immunocompromised status) or environmental circumstances (e.g., disruption of epithelial barriers). We also clarify that detection of such organisms by mNGS does not necessarily indicate clinical pathogenicity in the absence of supporting clinical or confirmatory diagnostic evidence. We have now included a note in this in the text (lines 187-189).

12) Line 121: I would phrase a different, more informative header, maybe mentioning the type of samples shown in the figure and the methods used rather than the tool used to identify them.

We have changed the figure title to “Microbial reads detected by mNGS in NP swabs from two cohorts”.

13) Line 122: use mNGS acronym

This change has been made, line 168.

14) Figure 1a: control 1/2 finding should be mentioned in the text (and defined better, also in methodology). E.g., it’s not clear to all readers that RSV, that’s included in the controls is a member of the Orthopneumovirus family. Also, it says in the figure legend “control 1-4” – that’s not clear. In addition, it says that just the 14 samples that were positive for bacteria are shown, but the figure legend says also “Rhinovirus” which is a virus. So, need to be more specific and clarify this in the text.

We apologize for the lack of clarity regarding the controls. We have now included a detailed explanation in the methods section to explicitly define controls 1–4 and revised the figure legend to clarify the identity of RSV. In addition, we have revised the text to make it explicit that the figure shows non-human sequences originating from viruses, bacteria, fungi or protists.

15) Figure 2 is not explained very well in the results section. Need to clarify what it shows and why it is important to show it as a main figure*.*

We thank the reviewer for this suggestion. The revised manuscript includes an explanation of Figure 2, including a description of the Pavian alignment viewer and its relevance for interpreting genome-level read coverage and assessing potential contamination or misclassification (lines 191-194).

16) Supp figure 1: not clear what e1-e6 is.

We have now edited the x-axis label to reflect the concentration of the dilution series, which ranged from 10⁶ to 10¹ copies/mL.

17) Line 144: the “controls” sub section can be integrated into the main text, each part where it fits, its missing from there (see my previous comments).

The authors appreciate the comment. We have now integrated this section into the relevant sections of the results as recommended to improve clarity and context.

**Reviewer #2:**

1) Table 01 – data not presented in a clear, legible manner. Why was data on race, ethnicity collected? Was there any analysis done relating to these factors? If not, does it add anything of value to the analysis?

The authors appreciate the comment; Table 1 has been revised and the race/ethnicity data omitted from the table.

2) Methods – the nucleic acid extraction mentions both RNA and DNA, but the library preparation only deals with RNA seq. was only RNA analyzed? In that case why was DNA also extracted?

We thank the reviewer for this comment. The ZR-Duet DNA/RNA miniprep used in this study enables extraction of purified RNA and DNA as separate fractions, which reduces DNA contamination in the RNA extract while also providing the option to analyze purified DNA. For this study, only the RNA fraction was utilized for library preparation and sequencing, as the metagenomic workflow was optimized for microbial RNA detection. We have now added this clarification in the revised manuscript (lines 117-121).

3) Controls – there seems to be a limitation on detection of Influenza A/B from Acrometrix positive control. Was this also tested on the Xpert Xpress/ePlex RP2 to check whether the targeted panels could detect as expected?

This a good question. Briefly, the Xpert Xpress CoV-2/Flu/RSV Plus and ePlex RP2 are FDA-authorized assays that include internal cartridge controls to ensure proper assay performance. In contrast, mNGS lacks standardized or regulatory recommended controls; therefore, a commercial positive control (Acrometrix™ Multi-Analyte SARS-CoV-2, influenza A/B, and RSV A/B), containing approximately 10⁴ copies/mL of each viral target was included to assess the mNGS workflow. In the absence of identical controls across platforms, findings were interpreted based on qualitative detection. This clarification has been added to the revised manuscript (lines 126-134).

4)Sequencing - can benefit from more detail on how much data per sample - this is a parameter that directly impacts limit of detection.

A median of 20,586,497 reads (IQR 14,78,026) was obtained from sequencing samples from Cohorts 1 and 2. This information has now been added to the revised manuscript on a cohort basis (lines 159-161,179-181). In addition, Supplementary Table 1 has been included to provide the read counts for each individual sample and control.

*5)*The details on how sensitivity/specificity was calculated is unclear. Would benefit from a specificity/sensitivity table.

Please see response to comment 6 below.

6) Overall conclusion needs more justification – you are saying NGS has high limit of detection but there is no data showing the ePlex and Xpert were run on the same controls. Using the clinical data for justification is difficult as there are variables such as storage time that may have impacted viability of RNA.

We thank the reviewer for this comment and have expanded the methods and discussion section to clarify. Briefly, the Xpert Xpress CoV-2/Flu/RSV Plus and ePlex RP2 are FDA-authorized multiplex molecular diagnostic assays that incorporate assay-specific internal controls within each cartridge to ensure proper sample processing and amplification. In contrast, unbiased metagenomic sequencing does not have standardized or regulatory-recommended control materials; therefore, external controls were incorporated specifically to assess the performance and analytical sensitivity of the mNGS workflow.

Our previous work on plasma demonstrated that when viral RNA levels measured by qPCR were <4 log_10_ copies or IU/mL, there was low correlation (r=0.28) between mNGS and qPCR. To better characterize the mNGS detection range in this study we included a dilution series ranging from 10⁶ to 10¹ copies/mL using a SARS-CoV-2–positive de-identified NP sample.

The reviewer also correctly highlights one of the study’s limitations that direct analytical sensitivity comparisons between mNGS and Xpert Xpress CoV-2/Flu/RSV Plus and ePlex RP2 could not be performed because identical external controls were not evaluated across platforms. Additionally, clinical sample variables such as storage duration may have affected RNA integrity, which could influence detection performance. Therefore, conclusions about mNGS performance are based on qualitative detection and clinical concordance rather than precise LOD comparisons

7)There should be more detail on the limitation of the study methodology, the NGS process etc. There is no analysis into the possible factors that may be affecting the detection or output on the NGS process.

We have added additional limitations to the discussion section to read ‘Other limitations include the sample volume processed for mNGS (200 µl), as processing larger sample volumes could increase the likelihood of sequencing low copy viral transcripts. Lastly, we only performed unbiased mNGS on these samples and utilization of one of the targeted sequencing panels that are commercially available could have produced different results. This has now been added to the revised manuscript (lines 235-239).

8) Figure 01 – It is not clear from the figure title that it is showing NGS data from a specific cohort.

We have now revised the figure title to **“*Microbial reads detected by mNGS in nasopharyngeal samples from two cohorts”*** to clarify that the data represent NGS results from these specific study cohorts.

9) Supplemental file – only ePlex detailed data is shared. Ideally the Xpert breakdown should also be included.

We thank the reviewer for this comment. Xpert results were not added to supplementary Table 2 because these samples were not tested with the Xpert assays. Additionally, all samples that were tested with Xpert were negative.
